# Timing of maternal death: Levels, trends, and ecological correlates using sibling data from 34 sub-Saharan African countries

**DOI:** 10.1371/journal.pone.0189416

**Published:** 2018-01-17

**Authors:** Leena Merdad, Mohamed M. Ali

**Affiliations:** 1 Department of Dental Public Health, Faculty of Dentistry, King Abdulaziz University, Jeddah, Saudi Arabia; 2 Department of Reproductive Health and Research, World Health Organization, Geneva, Switzerland; The Ohio State University, UNITED STATES

## Abstract

Millennium Development Goal 5 has not been universally achieved, particularly in sub-Saharan Africa. Understanding whether maternal deaths occur during pregnancy, childbirth, or puerperium is important to effectively plan maternal health programs and allocate resources. Our main research objectives are to (1) describe the proportions and rates of mortality for the antepartum, intrapartum, and postpartum periods; (2) document how these trends vary by sub-region; and (3) investigate ecological correlations between these rates and maternal care interventions. We used data from the Demographic and Health Survey program, which comprises 84 surveys from 34 sub-Saharan African countries conducted between 1990 and 2014. We calculated age-standardized maternal mortality rates and time-specific maternal mortality rates and proportions, and we assessed correlations with maternal care coverage. We found high levels of maternal mortality in all three periods. Time-specific maternal mortality rates varied by country and region, with some showing an orderly decline in all three periods and others exhibiting alarming increases in antepartum and postpartum mortality. Ecological analysis showed that antenatal care coverage was significantly associated with low antepartum mortality, whereas the presence of a skilled attendant at childbirth was significantly associated with low postpartum mortality. In sub-Saharan Africa, maternal deaths occur at high rates in all three risk periods, and vary substantially by country and region. The provision of maternal care is a predictor of time-specific maternal mortality. These results confirm the need for country-specific interventions during the continuum of care to achieve the global commitment to eliminating preventable maternal mortality.

## Introduction

In 2015, approximately 303,000 women died from complications of pregnancy and childbirth [1]. Most deaths (99%) occurred in low and middle income countries, with sub-Saharan Africa accounting for nearly two-thirds (66%) [1]. These statistics confirm that Millennium Development Goal 5 (reducing the maternal mortality ratio by three-quarters between 1990 and 2015) was not universally achieved, particularly in sub-Saharan Africa [2]. A renewed commitment to reducing maternal deaths was proposed in “*The Global Strategy for Women’s*, *Children’s and Adolescent’s Health*,” which has a vision of ending preventable maternal mortality, and in Sustainable Development Goal (SDG) 3, with a target 3.1 of reducing global maternal mortality to fewer than 70 per 100,000 live births by 2030 [3]. Achieving SDG 3.1 and ending preventable maternal mortality requires effective strategies and interventions to improve maternal health and enhance the monitoring and understanding of mortality trends [4].

Understanding the timing and causes of maternal deaths is essential to reducing maternal mortality, as it helps in planning health programs, setting priorities, and allocating resources (particularly necessary in sub-Saharan Africa, which has limited resources and dysfunctional health information systems). Unfortunately, accurate cause of death data—which is required for the identification of maternal deaths and their timing- is lacking in sub-Saharan Africa as a consequence of incomplete/non-existent vital registration systems. While there are myriad hospital-based studies on the timing of maternal deaths in sub-Saharan Africa [5,6], these studies are marred by selection bias and estimates that might not reflect the true maternal risk in the general population [7]. Prospective population-based studies have been conducted in several countries [8,9] to provide more accurate estimates; however, most were restricted to specific areas and thus may not provide nationally representative estimates.

To consolidate global data on the timing of maternal deaths, Kassebaum et al. [10] modeled the proportions of maternal deaths in four pregnancy-related periods using data from a systematic review of 142 studies and vital registration data. They found that, in 2013, nearly one-quarter of deaths occurred in the antepartum period (before onset of labor), another quarter occurred in the intrapartum and immediate postpartum periods (up to 24 hours after delivery), one-third occurred in the subacute and delayed postpartum periods (24 hours to 42 days after delivery), and 12% occurred in the late postpartum period (43 days to 1 year after delivery). Although intrapartum deaths have decreased by more than 35% since 1990, the estimates from sub-Saharan Africa showed fewer changes over time, and deaths in the postpartum period were over half of all deaths in each region of Africa [10]. Say et al. [11] similarly identified the global causes of maternal mortality between 2003–2009 from vital registration and bibliographic data and found that 72.5% were direct obstetric and 27.5% were indirect obstetric causes. Hemorrhage was the most common direct cause of maternal death globally (27%) and in sub-Saharan Africa (24%), and more than two-thirds of these deaths occurred in the postpartum period.

A continuum of maternal and newborn care during pregnancy, childbirth, and the postpartum period is essential for maternal and neonatal health [12]. The World Health Organization’s new guidelines on antenatal care (ANC) outline 49 evidence-based interventions to improve pregnancy outcomes [13]. Furthermore, skilled attendance at birth and emergency obstetric care have been promoted as strategies to reduce maternal mortality [14]. However, these efforts have not been rigorously evaluated for their effects on maternal mortality. Indeed, the role of skilled birth attendants in reducing maternal mortality was shown to be weak and mainly based on quasi-experimental [15], observational [16], or historical studies [17], and the efficacy of antenatal care in this regard has been questioned [18]. Although several ecological analyses have identified receiving antenatal care and having skilled birth attendants at birth as important determinants of maternal mortality in sub-Saharan African countries [16], none determined associations between these determinants and the timing of death.

To reduce maternal mortality in sub-Saharan Africa, investigating the timing of maternal deaths and the associations of maternal care with the timing of death is important. Although data from nationally representative surveys on the timing of maternal deaths have been available for many years, they have not been fully analyzed to inform actions in the SDG era. Thus, we present crucial results from a database of sibling histories drawn from 84 surveys in 34 sub-Saharan African countries. Our main research objectives are to (1) describe the proportions and rates of mortality for the antepartum, intrapartum, and postpartum periods; (2) document the trends in these rates and how they vary by sub-region; and (3) investigate the ecological correlations between these rates and maternal health care interventions.

## Data and methods

### Data source

We used data from Demographic and Health Surveys (DHS), which are national household surveys focusing on providing data on demographic and health issues in women of reproductive age (15–49 years). Data are collected from eligible women using a “woman’s questionnaire” to elicit information on these areas. Women are also asked to provide antenatal care and childbirth data about pregnancies that resulted in a live birth in the five years before the interview. The reference period was 3 years prior to the interview for some surveys conducted in the 1990s. A subset of these surveys contains a “Maternal Mortality Module” (i.e., sibling history), wherein respondents list every sibling born to their own mother and their survival status, sex, current age (if alive), and age and year of death (or years since death) if dead. For the deaths of sisters aged 15–50, additional questions are asked about the timing of death relative to pregnancy. Our analysis is based on data from 1990 to 2014 in 84 standard DHS [19] with a maternal mortality module from 34 sub-Saharan African countries (Annex 1). We divided countries into four regions—East, Central, South, and West—to provide a clearer picture of levels and trends.

### Estimating maternal mortality

Using survey data from the 84 DHS, maternal mortality was calculated as follows. First, age-specific maternal mortality rates (MMRates) were obtained by dividing the number of female sibling deaths (during pregnancy, childbirth, or two months postpartum) by the corresponding woman-years of exposure (WYE) in each age group. The overall MMRate (i.e., maternal deaths per 1,000 WYE for women aged 15–49) was then standardized using the 1990 United Nations (UN) age distribution of women aged 15–49 in sub-Saharan Africa [20]. We also estimated the proportions of maternal deaths and maternal mortality rates according to the timing of death (antepartum, intrapartum, and postpartum). All mortality indicators were estimated for the five-year period preceding the survey. Confidence intervals were estimated using bootstrapping. We employed the standard DHS approach for excluding respondents from the analysis and weighted data for female siblings using DHS sample weights.

### Associations between maternal care and maternal mortality

To investigate correlations between time-specific maternal mortality and maternal health care, we used DHS data on the number and timing of antenatal care visits as well as the presence of skilled birth attendants during childbirth. Skilled birth attendants were defined as medically trained and licensed personnel, including doctors, nurses, midwives, and auxiliary health personnel (or their professional equivalent in certain countries). We calculated the percentage of women who had 4 or more antenatal visits for their last birth and the percentage of women with births in the last five years who had their first antenatal care visit for their last birth in the first trimester (< 4 months). We also calculated the percentage of women whose childbirth was assisted by a skilled attendant during their last birth in the five years preceding the interview.

This analysis was based on each country’s most recent survey. Log-linear models were used to determine the relationship between use of antenatal care and antepartum, intrapartum, and postpartum mortality at the country level. A separate model was then fitted for each mortality rate. The predictors in each model were the percentage of women with 4 or more antenatal visits, the percentage of women with a skilled attendant at birth, gross national income (GNI), total fertility rate (TFR), and the percentage of women who had attended secondary school or higher. The World Bank publishes the annual GNI converted into international dollars using the purchasing power parity rates. We used the World Bank’s GNI per capita based on purchasing power parity [21]. For each country, linear interpolation was applied to the annual estimates to obtain an estimate for the midpoint of the 5 years preceding the survey. Information on the highest level of education attained or completed by women aged 15–49 was obtained from DHS data. The TFR is the average number of live births a woman would have if she were subject throughout her reproductive years (ages 15–49) to the current age-specific fertility rates. The TFR was calculated using DHS household data by adding the age-specific fertility rates (ASFRs) for the age groups of 15–19 to 45–49 years and then multiplying that value by five. The ASFRs were calculated by dividing the number of births in the 5 years preceding the survey to women in the corresponding age group at birth by the WYE for that age group. The regression coefficients, robust standard errors, and 95% confidence intervals are reported. STATA version 14 (StataCorp, College Station, Texas, USA) was used to analyze the data.

## Results

### Mortality indicators by timing of maternal death

All respondents and their reported siblings for each survey are displayed in Annex 3. Table 1 shows the age-standardized mortality rates and proportions of all maternal deaths for the antepartum, intrapartum, and postpartum periods for the most recent surveys. The MMRates ranged from 0.14 (0.08, 0.21) per 1000 WYE in South Africa in 1998 to 2.54 (2.06, 3.04) in the Central African Republic (CAR) in 1994–1995. Antepartum mortality ranged from 0.08 (95% CI: 0.03, 0.15) per 1000 WYE in South Africa to 0.77 (0.53, 1.03) in Sierra Leone; intrapartum mortality ranged from zero in Sao Tome and Principe to 1.33 (0.92, 1.74) in the CAR; and postpartum mortality ranged from 0.04 (0.00. 0.12) in Comoros to 0.78 (0.50, 1.12) in Lesotho. In 14, 11, and 9 of the 34 countries, the highest proportions of maternal deaths were in the postpartum, antepartum, and intrapartum periods, respectively.

**Table 1:** Percentages and age-standardized rates (per 1000 woman-years of exposure) of maternal mortality by timing of death (antepartum, intrapartum and postpartum) for the five years preceding the survey.

Country	Year	Maternal Deaths	MMRate	Antepartum		Intrapartum		Postpartum	
				%	Rate	%	Rate	%	Rate
*East Africa*									
Burundi	2010	64	0.87	32.5	0.26	24.3	0.20	43.2	0.41
Comoros	2012	16	0.31	44.4	0.15	39.5	0.12	16.1	0.04
Ethiopia	2011	128	0.94	39.0	0.35	37.1	0.35	23.9	0.24
Kenya	2008–09	61	0.75	35.8	0.28	25.4	0.20	38.8	0.27
Madagascar	2008–09	136	0.92	44.8	0.41	31.3	0.29	23.9	0.22
Malawi	2010	223	1.30	22.7	0.31	48.6	0.62	28.7	0.36
Mozambique	2011	88	0.90	64.8	0.60	9.7	0.09	25.5	0.22
Rwanda	2010	91	0.72	25.0	0.17	31.4	0.24	43.6	0.31
Tanzania	2010	81	0.85	30.6	0.27	25.1	0.22	44.3	0.37
Uganda	2011	64	0.79	30.9	0.24	27.5	0.20	41.6	0.34
Zambia	2007	75	1.18	31.2	0.34	24.5	0.33	44.3	0.50
Zimbabwe	2010–11	113	1.36	38.3	0.51	20.3	0.28	41.4	0.57
*Central Africa*									
CAR	1994–95	120	2.54	23.1	0.55	51.3	1.33	25.5	0.67
Cameroon	2011	177	1.30	41.9	0.54	21.9	0.27	36.2	0.49
Chad	2004	111	2.25	27.8	0.60	43.3	0.96	28.9	0.69
Congo (Brazzaville)	2011–12	66	0.71	39.8	0.30	49.9	0.34	10.3	0.07
CDR	2013–14	300	1.77	41.9	0.75	34.6	0.61	23.5	0.41
Gabon	2012	23	0.38	57.0	0.24	28.7	0.09	14.3	0.05
Sao Tome and Principe	2008–09	5	0.23	47.6	0.09	0.0	0.00	52.4	0.13
*South Africa*									
Lesotho	2009–10	77	1.29	25.6	0.34	12.9	0.17	61.6	0.78
Namibia	2013	29	0.37	49.1	0.18	25.4	0.08	25.5	0.11
South Africa	1998	13	0.14	56.5	0.08	6.0	0.01	37.5	0.05
Swaziland	2006–07	43	0.99	43.4	0.43	14.5	0.14	42.2	0.42
*West Africa*									
Benin	2006	114	0.75	31.3	0.24	35.4	0.27	33.3	0.24
Burkina Faso	2010	95	0.65	27.0	0.16	22.1	0.13	50.9	0.35
Cote d’Ivoire	2011–12	88	1.08	28.2	0.28	38.4	0.42	33.5	0.38
Guinea	2012	84	1.33	32.9	0.42	32.9	0.45	34.1	0.45
Liberia	2013	142	1.81	31.1	0.57	40.8	0.71	28.1	0.53
Mali	2012–13	61	0.79	36.6	0.30	32.4	0.26	31.0	0.23
Niger	2012	156	1.59	45.0	0.71	18.5	0.30	36.6	0.58
Nigeria	2013	343	1.17	42.9	0.52	37.2	0.42	20.0	0.24
Senegal	2010–11	111	0.76	26.9	0.19	36.5	0.28	36.6	0.29
Sierra Leone	2013	217	2.17	35.1	0.77	43.6	0.95	21.4	0.45
Togo	1998	58	0.77	42.2	0.30	16.0	0.11	41.8	0.36

Abbreviations: MMRate, maternal mortality rate

The maternal mortality rate is measured as maternal deaths per 1,000 woman-years of exposure.

**Fig 1:**
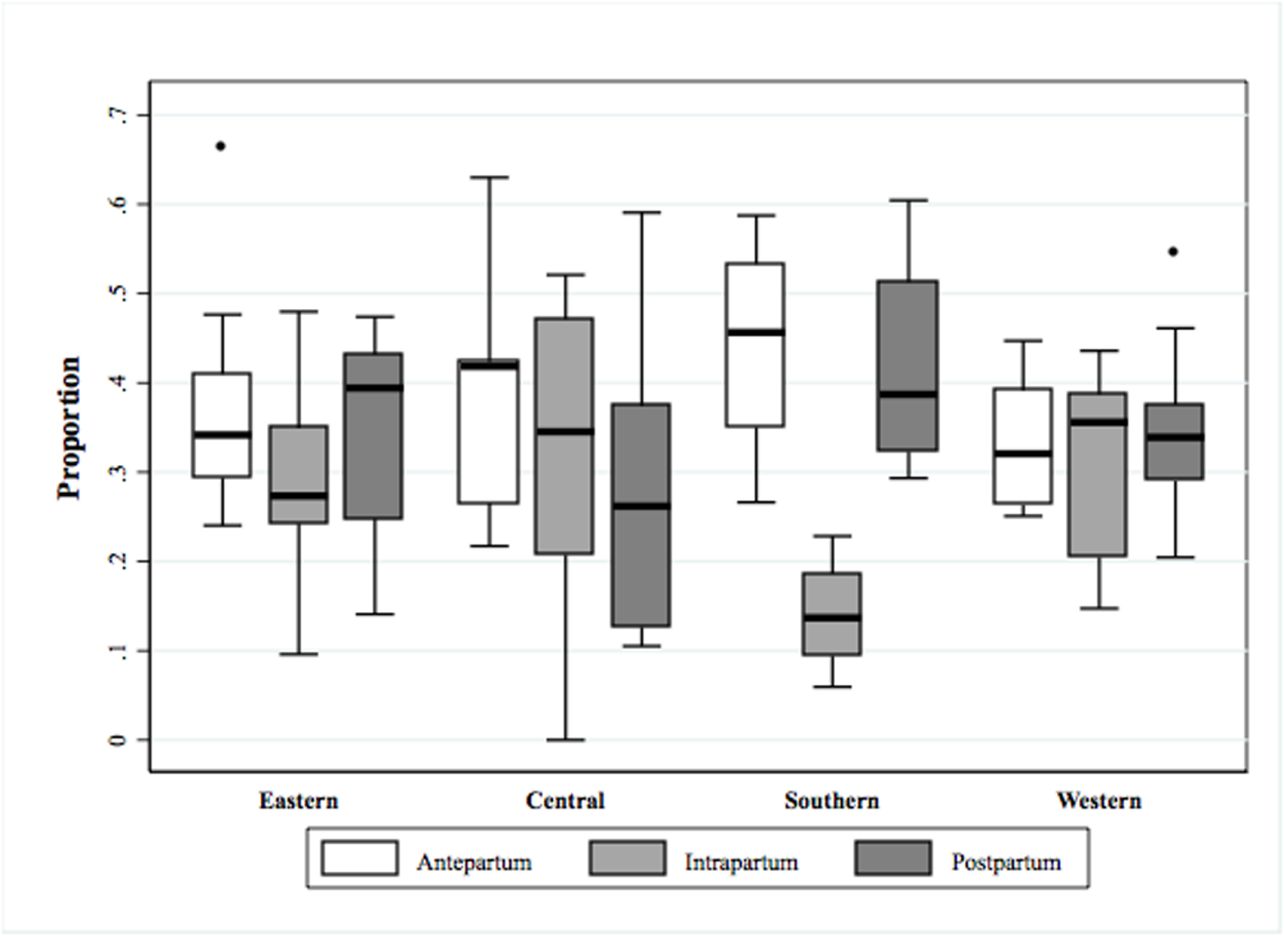
Timing of maternal death (antepartum, intrapartum and postpartum) by sub-Saharan African region.

In most countries, maternal deaths occurred mainly in the antepartum and postpartum periods; however, there were notable variations in the distributions of the timing of death within and among sub-Saharan African regions (Fig 1). In East Africa, postpartum deaths constituted the highest proportion of maternal deaths (median = 0.39, interquartile range [IQR]: 0.25–0.43) followed by antepartum (median = 0.34, IQR: 0.29–0.41) and intrapartum deaths (median = 0.27, IQR: 0.24–0.35). In Central Africa, the proportions of antepartum (median = 0.42, IQR: 0.27–0.42) and intrapartum deaths (median = 0.34, IQR: 0.21–0.47) were higher than the proportion of postpartum deaths (median = 0.26, IQR: 0.13–0.37). In South Africa, the proportion of antepartum deaths (median = 0.46, IQR: 0.35–0.53) was highest, followed by the proportions of postpartum (median = 0.39, IQR: 0.32–0.51) and intrapartum deaths (median = 0.14, IQR: 0.09–0.19). However, in West Africa, the highest proportion was for intrapartum deaths (median = 0.36, IQR: 0.20–0.39), followed by postpartum (median = 0.34, IQR: 0.29–0.38) and antepartum deaths (median = 0.32, IQR: 0.26–0.39). The proportions of antepartum, intrapartum, and postpartum deaths and age-standardized maternal mortality rates and their confidence intervals for all countries are listed in Annex 4.

### Trends in the timing of maternal mortality indicators

**Fig 2:**
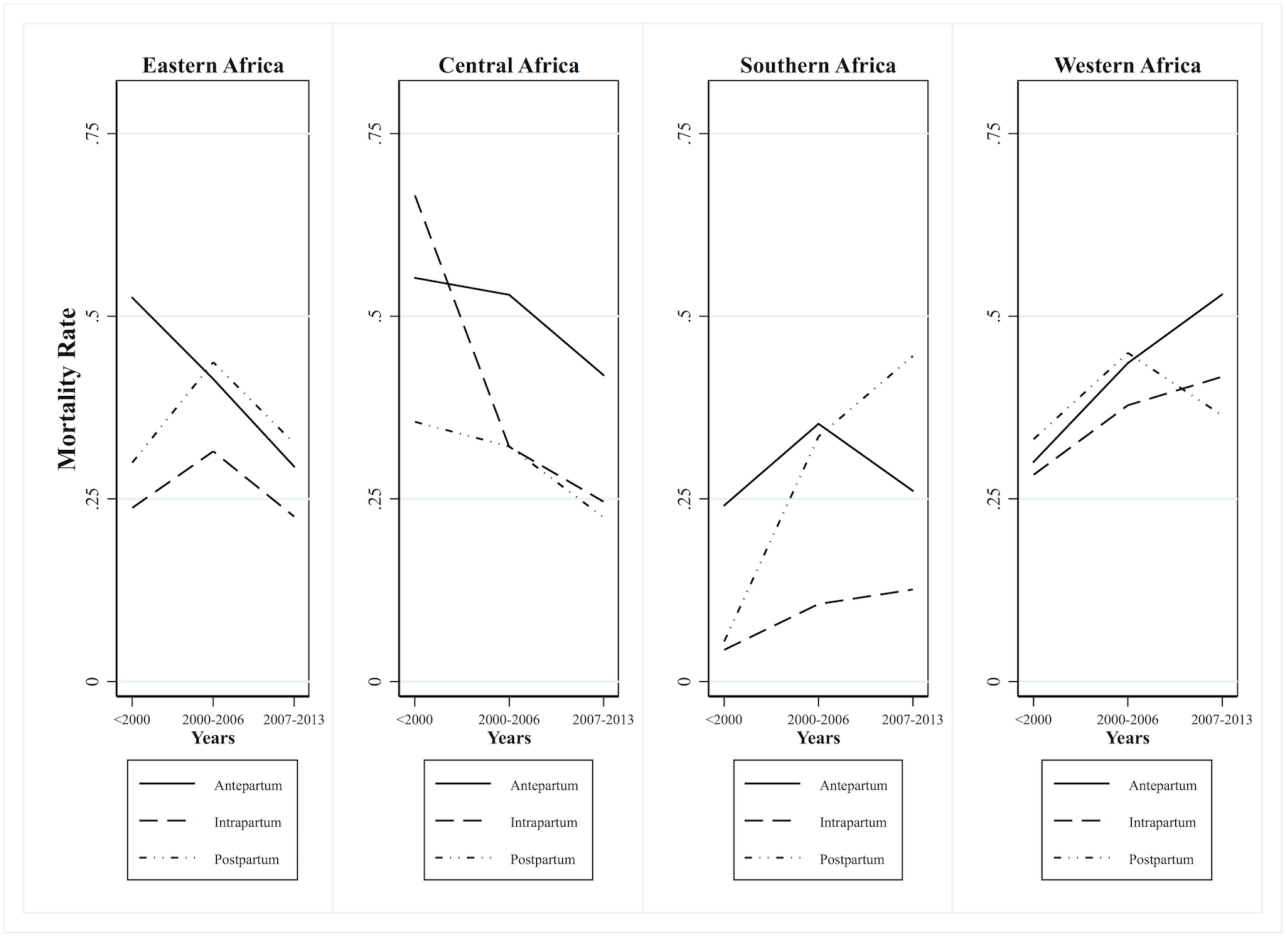
Trends in timing of maternal death (antepartum, intrapartum and postpartum mortality rates) by sub-Saharan African region.

Fig 2 illustrates the trends in antepartum, intrapartum, and postpartum mortality rates for all countries in each sub-Saharan African region. We observed a decrease in antepartum mortality in East Africa, while Central African countries showed a substantial decrease in intrapartum mortality and, to a lesser extent, antepartum and postpartum mortality. Conversely, South Africa showed an increasing trend in intrapartum and postpartum mortality and West Africa showed an increase in antepartum and intrapartum mortality.

### Maternal health care coverage

**Fig 3:**
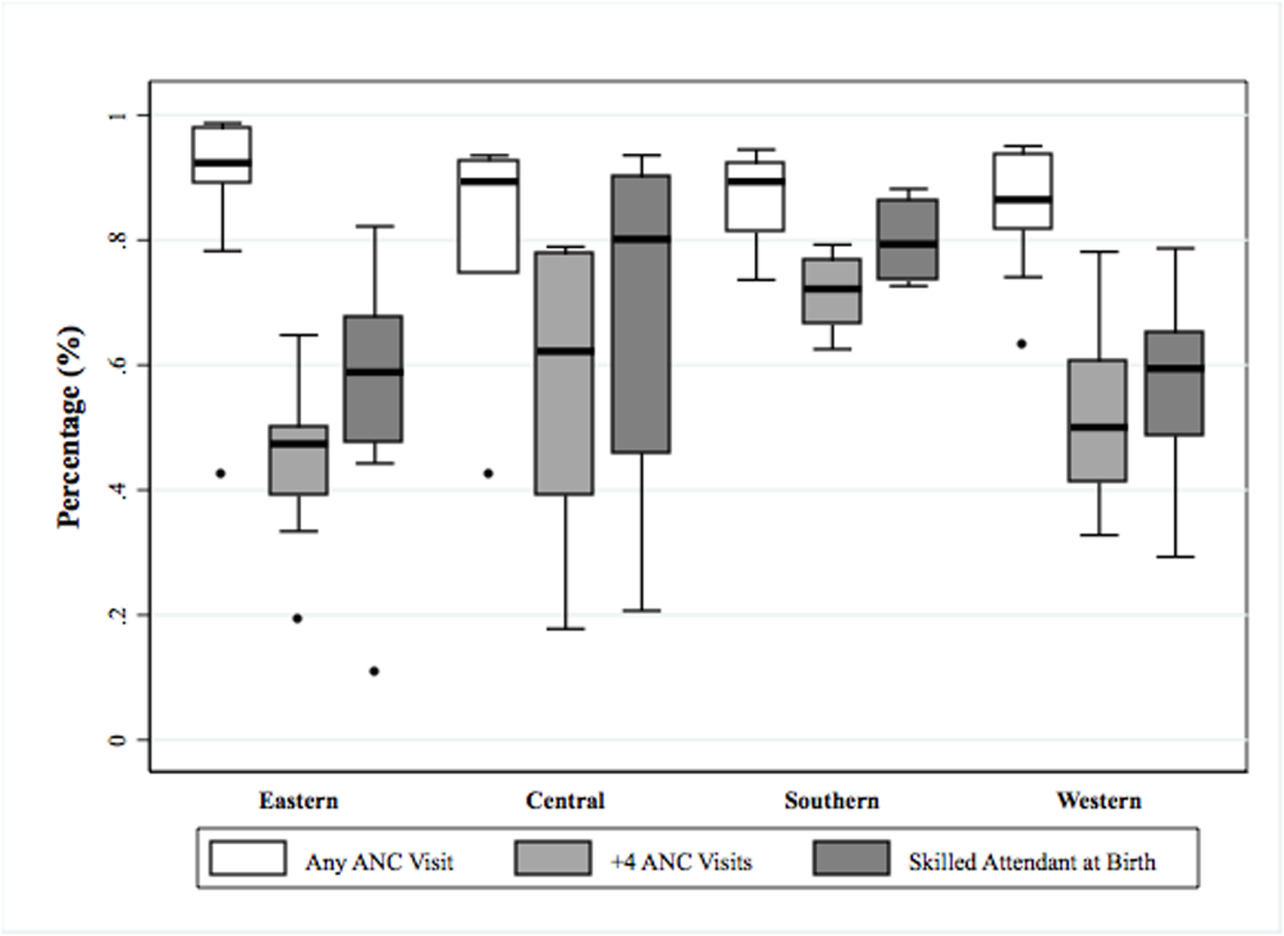
Antenatal and childbirth care by sub-Saharan African region.

The proportions of women making at least one antenatal care visit and four or more antenatal care visits for the last live birth in the five years before the interview and the proportion of women who used a skilled birth attendant for their last birth are shown in Annex 5. Fig 3 shows the proportions of women with at least one or at least four antenatal care visits and who had used a skilled attendant in each region of sub-Saharan Africa. Antenatal coverage was generally high in all regions (median = 0.90, IQR: 0.84–0.94). The proportion of women with at least four visits was highest in South Africa, followed by Central, West, and East Africa. A similar pattern was observed for the presence of skilled birth attendants.

### Maternal health care and timing of maternal death

**Fig 4:**
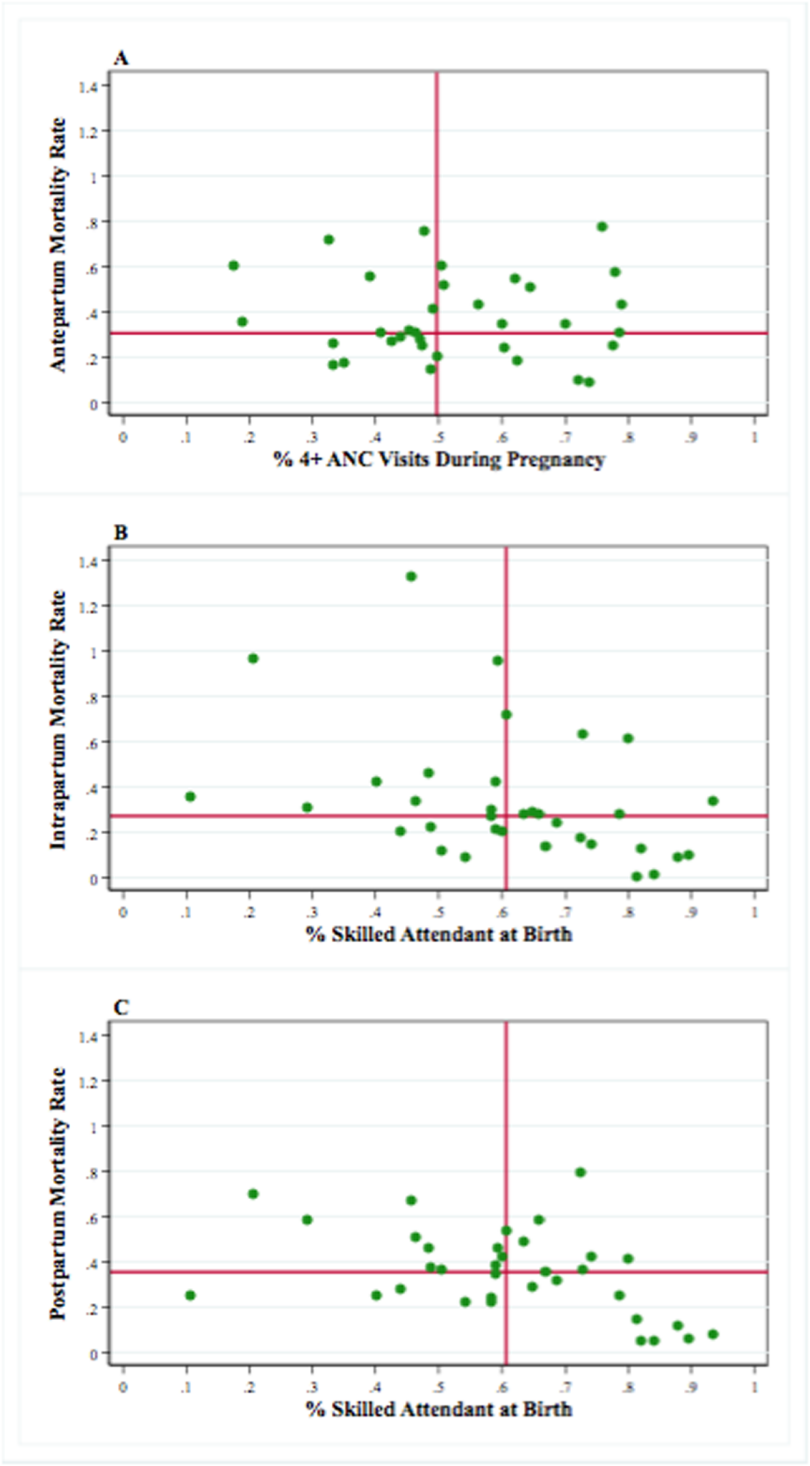
Timing of maternal death (antepartum, intrapartum and postpartum mortality rates) versus antenatal care and childbirth. (A) Antepartum mortality vs. number of ANC (antenatal care) visits; (B) Intrapartum vs. skilled attendant at birth; (C) Postpartum mortality vs. skilled attendant at birth.

Fig 4 presents the associations between the timing of death and antenatal care and the presence of skilled attendants at childbirth. The results presented in Table 2 display the coefficients and 95% confidence intervals from the ecological log-linear models evaluating the association of antenatal care and skilled birth attendants with antepartum, intrapartum, and postpartum mortality rates. We found that antenatal care was significantly associated with lower antepartum mortality. Skilled birth attendants were not associated with intrapartum mortality but were associated with lower postpartum mortality.

**Table 2:** Coefficients, standard errors and 95% confidence intervals for predictors of timing of maternal death (antepartum, intrapartum and postpartum mortality rates).

Timing of Maternal Death	Predictors	Coefficient	95% Confidence Intervals	
Antepartum mortality	4+ ANC visits	-0.87	-1.65	-0.10
	TFR	0.21	0.06	0.36
	Education	1.35	0.56	2.13
	log GNI	-0.35	-0.58	-0.12
Intrapartum mortality	SBA	-0.82	-2.08	0.425
	TFR	-0.10	-0.38	0.185
	Education	0.32	-1.62	2.259
	log GNI	-1.00	-1.72	-0.286
Postpartum mortality	SBA	-0.95	-1.80	-0.09
	TFR	0.01	-0.18	0.20
	Education	0.84	-0.43	2.11
	log GNI	-0.39	-0.67	-0.11

Abbreviations: ANC, antenatal care; SBA, skilled birth attendant; TFR, total fertility rate; GNI, gross national income

## Discussion

Our analysis of DHS from 1990 to 2014 for 34 countries provides new evidence on the levels and trends in the timing of maternal death in sub-Saharan Africa. High levels of maternal mortality were shown during the three periods. The results also show that time-specific maternal mortality rates differed widely by country and region; in some, we observed an orderly decline in mortality for all three periods, while in others, alarming increases were observed. Furthermore, the ecological analysis revealed that antenatal care was significantly associated with lower antepartum mortality, while skilled birth attendants were associated with lower postpartum mortality.

The main causes of maternal deaths can be divided by when they occur. The main reasons for deaths in the antepartum period include ectopic pregnancy, abortion and antepartum hemorrhage as well as hypertensive disorders that continue throughout labor and the early puerperium; those in the intrapartum period include complications of obstructed labor, hemorrhage and other childbirth complications; and those in the postpartum period include hemorrhage and sepsis. Indirect causes, such as HIV, malaria and pre-existing medical conditions, are also important and account for approximately 29% of maternal deaths in sub-Saharan Africa [22].

As in a recent DHS assessment of maternal mortality data [23], we observed high maternal mortality in all three periods, including the antepartum period, and often the three periods were equally important. However, we observed substantial heterogeneity among countries, with occasional departures from this pattern in countries where intrapartum or postpartum mortality dominated, for example, CAR in 1994–95 and Cote d’Ivoire in 2011–12. Antepartum mortality was higher than would be expected from previous studies, in which maternal deaths are commonly reported as occurring in the intrapartum or postpartum periods [10]. Similarly, hospital-based studies conducted in sub-Saharan Africa report increased risks during those periods [5,6] (these studies, however, have their limitations, as noted in the introduction) [7]. Several population-based studies have estimated the proportion of antepartum deaths to markedly differ by country, ranging from as low as 4.7% in Sudan [24] to as high as 31.5% in Nigeria [8]. Additionally, several population-based studies showed a higher mortality rate soon after childbirth that declined sharply over subsequent weeks [9,25].

The high antepartum mortality and the heterogeneity among countries and regions in the distribution of timing of maternal deaths may be a reflection of the true population distribution of underlying causes, but it might also be a result of inaccurate data, the inclusion of incidental deaths or the variation in the proportion of deaths attributable to abortion. In their assessment of DHS maternal mortality data, Ahmed et al. found that the distribution of timing of maternal death was similar across surveys from the same country and across different recall periods in the same survey [23]. However, a record linkage study in Senegal showed that information on sibling survival often suffered from reporting errors, particularly omitting deceased or emigrated sisters and underestimating the age at death of sisters who had died at older ages [26]. Thus, the respondents’ ability to provide an accurate survival history, especially those with little contact with their sisters at the time of their deaths, is a cause of concern. Additionally, accurate reporting of the timing of death may be problematic around the time of childbirth, and perceptions might vary on how the deaths should be classified. For example, a sister dying from an antepartum hemorrhage could be reported as a “death during pregnancy” as much as a “death during childbirth.” Differences in the proportions of incidental deaths included might also influence the distribution of the timing of death. Finally, abortion-related deaths (which are normally classified as antepartum deaths) are difficult to measure and estimate [27], and variations in the proportion of such deaths are also likely to be a factor.

In line with previous studies on the general global trends in maternal mortality, including sub-Saharan Africa, we observed widely differing trends across countries and regions [2,10]. We observed an orderly decline in all rates in Central and East Africa but increases in intrapartum and postpartum rates in South Africa and antepartum and intrapartum rates in West Africa. Although we cannot provide explanations for these trends, general declines in maternal mortality have been explained by correlates such as fertility, educational attainment, and income; increases have been explained by HIV epidemics [10]. How these correlates influence maternal deaths in each period requires further investigation. Furthermore, international assistance should focus on countries in South and West Africa, where mortality remains high.

Countries with high antenatal care coverage had lower antepartum mortality. Although a recent systematic review reported that fewer but more goal-oriented antenatal visits had no impact on maternal mortality, the quality of the reviewed studies suffered from design limitations and imprecision due to studies being underpowered [18]. Another important finding is that antenatal care is measured in terms of frequency of visits, which provides no information on the content, quality, timing, or access to visits. Thus, a more comprehensive measurement of ANC is recommended [28,29].

The presence of a skilled attendant at childbirth was linked with lower postpartum but not intrapartum mortality. Given that the immediate postpartum period is linked clinically to events occurring during labor and childbirth, we expect that the benefits of labor and childbirth interventions, such as the presence of skilled birth attendants, would extend into the postpartum period. The effects were not visible in the intrapartum period because it is relatively short, and it is more challenging to provide adequate care during this period, particularly in the many sub-Saharan African countries with poor health systems and human resources shortages, which constrain the availability of emergency obstetric care [30]. Improving the skills and number of skilled birth attendants and implementing a health care human resources management system are necessary to reduce maternal mortality in these countries. Meanwhile, based on current evidence, maternal care should be integrated into community settings—for instance, community health workers could effectively deliver interventions to reduce maternal morbidity and possibly mortality [31].

Our study included a large number of surveys from different countries across sub-Saharan African regions. Using standardized mortality rates enabled comparisons across countries. The availability of data on timing-specific mortality and maternal care in the same country’s surveys enabled the assessment of the association between these factors. However, this study has several limitations. First, the quality of the timing of death data—collected through sibling histories—and the likelihood of misreporting errors are of concern. Interviews with families who were living with the woman at the time of her death might provide more accurate information about the timing. Second, the lack of consistency among studies in how the risk periods are defined makes direct comparisons to our results difficult, particularly around the time of labor. For instance, some studies use the cut-off of “before the end of pregnancy” for the antepartum period, which includes women who die in labor and who did not deliver, while others used “before the onset of labor.” For the intrapartum period, some studies combined the immediate or early postpartum period (first 24 hours postpartum) with the intrapartum period. Fourth, information on numerous determinants of maternal mortality is not collected by the DHS sibling histories, which precludes conducting a more in depth analysis of factors associated with maternal deaths. Finally, the association between maternal care and time-specific maternal mortality was based on an ecological analysis; therefore, the results cannot be interpreted as causal and conclusions cannot be applied to individual women.

## Conclusion

Maternal mortality in sub-Saharan Africa is high, with substantial mortality during all three risk periods, including the antepartum period. Time-specific maternal mortality showed a general decline in all three periods but alarming increases in intrapartum and postpartum rates in South Africa and antepartum and intrapartum rates in West Africa. There is substantial heterogeneity among countries and regions in the levels and trends of time-specific mortality. The receipt of maternal health care may be an important determinant of the timing of maternal deaths.

There is an obvious need for better data on why maternal deaths occur. Sibling histories aid in filling the gap left by incomplete civil registration systems but further research is needed to investigate the use of new data collection tools like verbal autopsy instruments that might help better ascertain the number and causes of maternal death. In addition, the continuous investment in the strengthening of vital registration systems is essential.
